# Nyctalopia during pregnancy due to vitamin A deficiency after bariatric sleeve gastrectomy: A case report

**DOI:** 10.1002/ccr3.9387

**Published:** 2024-09-06

**Authors:** Eleftherios Anastasakis, Anastasios Anastasakis, Athina A. Samara, Antonios Koutras, Zacharias Fasoulakis, Athanasios Zikopoulos, Chara Skentou, Sotirios Sotiriou

**Affiliations:** ^1^ Department of Obstetrics and Gynecology Iaso Maternity Hospital Athens Greece; ^2^ Department of Ophthalmology Athens Eye Hospital Athens Greece; ^3^ Department of Embryology, Faculty of Medicine University of Thessaly Larissa Greece; ^4^ Department of Obstetrics and Gynaecology Alexandra General Hospital Athens Greece; ^5^ Department of Obstetrics and Gynaecology Royal Cornwall Hospital Truro UK; ^6^ Department of Obstetrics and Gynaecology, Medical School University of Ioannina Ioannina Greece

**Keywords:** gastrectomy, malabsorption, morbid obesity, night vision, vitamin A

## Abstract

**Key Clinical Message:**

Herein, we report a rare case of nyctalopia diagnosed in the first trimester of pregnancy due to vitamin A deficiency as a result of a bariatric gastrectomy. Low serum vitamin A levels establish the diagnosis and the patient was treated with oral vitamin A supplements. Moreover, due to the teratogenic effects of exceed Vitamin A levels in early pregnancy, supplements' dosages should be prescribed with respect to the safe limits. Our case aims to highlight the importance of checking micronutricients and vitamins levels before and during pregnancy in women that had a previous bariatric surgery.

**Abstract:**

Vitamin A deficiency (VAD) has been identified as the predominant factor in the development of night blindness during pregnancy, a high‐risk for morbidity situation. Herein, we report a rare case of nyctalopia diagnosed in the first trimester of pregnancy due to VAD as a result of a bariatric gastrectomy. Our case aims to highlight the importance of checking micronutricients and vitamins levels before and during pregnancy in women that had a previous bariatric surgery. Low serum vitamin A levels establish the diagnosis and the patient was treated with oral vitamin A supplements. An uneventful antenatal course resulted in the birth of a healthy live neonatal at 38 weeks of gestation. In conclusion, nyctalopia is a rare condition in pregnant women that is often caused by VAD that poses significant health risks for both the mother and the infant, especially in women with a history of gastrointestinal bypass surgery, or any factors leading to malnutrition. Clinicians have to be alerted for micronutrients deficient in pregnant women who have a bariatric operation in their medical history.

## 1 INTRODUCTION

Nyctalopia, also known as moonblink, refers to night blindness or difficulty of the eye in visualizing under dim light or at night, with daytime vision remaining unimpaired. Nyctalopia is caused by the eye's inability to adapt quickly from lightness to darkness.^1^ This condition could be the first presenting symptom of inherited conditions such as retinitis pigmentosa or acquired diseases such as vitamin A deficiency (VAD), as night blindness is sensitive and specific for serum retinol levels and is the earliest clinical manifestation of VAD.^2^ Myopia, or nearsightedness, is refractive error pathology that can also cause nyctalopia.^3^

VAD has been identified as the predominant factor in the development of night blindness during pregnancy.^4^ Vitamin A, also called retinol, is a fat‐soluble vitamin crucial for both the pregnant woman and the developing fetus. Retinol is essential for ocular metabolism in adults and exerts systemic effects on several fetal organs and the fetal skeleton. Vitamin A is a necessary component for the synthesis of visual pigments in rods and cones that also plays a role in photo signal transduction, as well as in the maintenance of epithelial surfaces on the cornea and conjunctiva.^4^ VAD is commonly the result of malabsorption, malnutrition or a result of liver disease. It may lead to significant dysfunction at the level of retinal photoreceptors, mainly on the rod system, that is responsible for night vision.^5^

Night blindness during pregnancy caused by VAD is associated with an increased risk of morbidity among women and children.^6^ Furthermore, studies among infants in Nepal have indicated that maternal nyctalopia increases the risk of mortality in the first 6 months of life.^7^ Moreover, nyctalopia during pregnancy has been linked to low birth weight, morbidity, and poor growth in infants.^8^ Vitamin A is important for the fetal development as it is a necessary element for visual acuity, immunity, bone growth, and epithelial cell differentiation.^9^

Herein, we report a rare case of nyctalopia during pregnancy due to VAD as a result of a bariatric gastrectomy. Our case aims to highlight the importance of checking micronutricients and vitamins levels before and during pregnancy in women that had a previous bariatric surgery.

## 2 CASE HISTORY

A 36‐year‐old primigravida Caucasian female presented during the first trimester of pregnancy (12th week of gestation) complained for reduced vision, that occurs mainly during the evening, while she reported normal vision in the day light. Her symptoms began a week ago and the patient experienced anxiety and forced to limit her social life and due to the affected night vision and disability to drive her car.

The pregnancy was uneventful so far and the conceive was a result of in vitro fertilization (IVF). Her medical history included Systemic Lupus Erythematosus for which she was receiving methotrexate for the last 12 years, hypothyroidism under treatment with thyroxine, and a laparoscopic bariatric sleeve gastrectomy to treat morbid obesity. Her obstetric medical history included eight previous failed IVF attempts and her Body Mass Index (BMI) was calculated in normal ranges.

She was referred to the ophthalmologist who recorded visual acuity 6/6 in each eye and additionally, electrophysiological tests were performed to objectively assess visual function. Rod system responses, representing scotopic function, were found severely reduced while cone system responses, representing photopic function, were found normal. Moreover, the patient reported an uneventful first trimester of gestation without any nausea and vomiting.

## 3 METHODS

Taking into consideration her past medical history (sleeve gastrectomy) the working diagnosis included, acquired causes of night blindness, such as VAD. Serum vitamin A levels were requested revealing reduced serum vitamin A levels (0.17 mg/L, with reporting normal range 0.30–0.60 mg/L).

Subsequently based on the World Health Organization (WHO) recommendations, oral vitamin A supplementation for 4 weeks (10.000 IU daily until delivery) were administrated, resulted in complete restoration of visual function and normalization of scotopic responses on electrophysiologic testing after the first 2 weeks of replacement therapy.

## 4 CONCLUSION AND RESULTS

Serum levels of Vitamin A were measured at normal ranges during the 20th and 32nd weeks of gestation. An uneventful antenatal course resulted in the birth of a healthy live neonatal at 38 weeks of gestation.

## 5 DISCUSSION

Vitamin A requirements during pregnancy are increased (370 mg/day), compared to non‐pregnant woman (300 mg/day).^10^ The WHO defines deficiency in pregnancy as serum retinol levels of <0.70 μmol/L. However, the role of serum retinol as a marker of vitamin A status during pregnancy has been questioned, as the relation between hepatic reserves and circulating retinol is altered due to the physiological changes during pregnancy.^11^ In general, when there is no deficiency, Vitamin A is stored in the liver in order to cover extra needs. Hepatic reserves and circulating retinol relationship is altered due to the physiological changes during pregnancy, especially during the third trimester. Moreover, retinol‐binding protein, the retinol transporter protein in the serum, is an acute phase protein and its concentration may differ in inflammatory situations.^11^ During gestation an inflammatory process in view of the immunological adaptations necessary to ensure the viability of the conceptus is physiologically occurring.^12^ Thus, serum Vitamin A levels and its conventional cut‐off point may be underestimating the status of vitamin A during the last trimester of pregnancy.^10^ Due to the potential teratogenic effects secondary to excessive vitamin A intake, the WHO recommends as safe during pregnancy a maximum dose of up to 10,000 IU daily or 25,000 IU weekly after the first 60 days of gestation.^10^

Routine prenatal and antenatal vitamin A supplementation is not recommended; however, in regions where VAD is a public health issue, mainly in developing countries, supplementation is recommended to prevent night blindness.^10^ High intakes of vitamin A (>3000 μg/day or >10,000 IU/day) during pregnancy are associated with an increased risk of malformations and should be avoided.^13^ In the current case, low serum vitamin A levels at the 12th week of gestation allowed us to establish the diagnosis and begin treatment with oral vitamin A supplements, with no concerns regarding the possible teratogenic effects that this micronutrient can exert in the first 60 days following conception.

Pregnancy in women with previous bariatric surgery needs special considerations. Weight loss after a bariatric operation decrease the perinatal and pregnancy risks. However, due to its effect on nutrient intake and absorption, it can also have adverse consequences on maternal and fetal health. Among them, vitamin and mineral deficiencies are especially frequent and require a protocolised evaluation and treatment.^13^ The risk of mineral and micronutrient deficiencies following an operation for morbid obesity is associated with the patient's dietary intake and on the iatrogenic anatomical and functional modifications following the surgery per se. In cases where techniques that exclude the duodenum and first jejunal loops or with a long biliopancreatic limb malabsorption may be a frequent complication.^13^

One of the leading causes of clinical significant VAD nowadays in developed countries is bariatric operations, and especially in cases where malabsorptive techniques are applied, the prevalence of decreased vitamin A serum levels can be as high as 60%.^14^ In less radical techniques VAD is considered less frequent and in gastric bypass surgery, a prevalence of 11% has been reported, associated with visual symptoms such as xerophthalmia or night vision impairment.^15^ Postoperative VAD following a bariatric surgery has been linked with unfavorable effects on pregnancy.^16^ Cases of malformations, such as microphthalmia, and other fetal complications secondary to maternal VAD have been reported. The first case of clinical VAD during gestation was published in 2002 describing a VAD case 13 years after the biliopancreatic diversion. Since then, several cases of VAD as a consequence of bariatric surgeries were published.^17^, ^18^, ^19^

As up to 80% of bariatric operations are performed in women, with the half of them of reproductive age, following the recommendations of dietary intake of nutrients and micronutrients, both during the pre‐conception period and throughout the pregnancy, is essential in order to avoid adverse perinatal outcomes. Women in childbearing age, undergoing bariatric procedures should be provided with appropriate counseling about adequate contraception, the optimal recommended time‐to‐conception interval, and the positive and negative influence of bariatric surgery on perinatal outcomes.^20^ The goal of sleeve gastrectomy is the decrease of stomach volume by removing a large part of the stomach, without bypassing any part of the digestive tract. Considering the fact that the main cause of malabsorption following a bariatric procedure is the bypassing of parts of ileum and jejunal, signs of malabsorption after a sleeve gastrectomy is a rare event. However, preoperative mild VAD can be associated with post bariatric surgery VAD even in restrictive operations, such as sleeve gastrectomy.^21^ Moreover, albumin levels, correlated with nutrition status, are strongly linked with serum vitamin A levels.^21^ Preoperative assessment of Vitamin A levels before bariatric surgery, alongside with measurements prior to pregnancy attempts could prevent gestational VAD.

In conclusion, nyctalopia is a rare condition in pregnant women that is often caused by VAD. VAD poses significant health risks for both the mother and the infant, especially in women with a history of a bariatric surgery and other factors leading to malnutrition. Clinicians have to be alerted for micronutrients deficient in pregnant women with a bariatric operation in their medical history. Moreover, due to the teratogenic effects of exceed Vitamin A levels in early pregnancy, supplements' dosages should be prescribed with respect to the safe limits.

## AUTHOR CONTRIBUTIONS

**Anastasios Anastasakis:** Conceptualization; methodology; writing – original draft. **Eleftherios Anastasakis:** Conceptualization; investigation; methodology. **Athina A. Samara:** Conceptualization; writing – original draft. **Zacharias Fasoulakis:** Investigation; methodology. **Antonios Koutras:** Investigation; methodology. **Athanasios Zikopoulos:** Investigation; methodology. **Chara Skentou:** Investigation; supervision. **Sotirios Sotiriou:** Conceptualization; methodology; supervision.

## FUNDING INFORMATION

That study received no funding.

## CONFLICT OF INTEREST STATEMENT

Authors declare no conflict of interest.

## CONSENT

A written informed consent was obtained from the patient to publish this report in accordance with the journal's patient consent policy.

## Data Availability

The data that support the findings of this study are available on request from the corresponding author. The data are not publicly available due to privacy or ethical restrictions.
